# The effect of neoadjuvant chemotherapy on the tumor immune microenvironment in gastrointestinal tumors

**DOI:** 10.3389/fonc.2022.1054598

**Published:** 2022-11-08

**Authors:** Yujie Wang, Peng Gao, Zhibin Hao, Ling Chen, Xiaoxiao Li, Yuan Jiao, Jingyu Liu, Jie Li, Yingyi Zhang, Xiaobo Peng, Beifang Ning, Xianbao Zhan

**Affiliations:** ^1^ Department of Oncology, Changhai Hospital, Naval Military Medical University, Shanghai, China; ^2^ Clinical Cancer Institute, Center for Translational Medicine, Naval Military Medical University, Shanghai, China; ^3^ Department of Gastroenterology, Changzheng Hospital, Naval Military Medical University, Shanghai, China

**Keywords:** tumor immune microenvironment, tumor-infiltrating immune cells, neoadjuvant chemotherapy, immunotherapy, prognostic value

## Abstract

In recent years, numerous studies have demonstrated that the tumor immune microenvironment (TIME) is capable of regulating the growth of tumors, and tumor-infiltrating immune cells in the TIME can affect the prognosis and treatment responses of patients. Consequently, therapies targeting these immune cells have emerged as important antitumor treatments. As a crucial componet of the perioperative treatment of malignant tumors, neoadjuvant chemotherapy (NACT) can improve the surgical resection rate and prognosis of patients and is a suitable clinical model to evaluate the effect of chemotherapy on the TIME. To provide a rationale for developing valid combinational therapies, this review summarizes the impact of NACT on the TIME, the relationship between tumor-infiltrating immune cells and treatment responses of patients, and the prognostic value of these infiltrating immune cells.

## Introduction

Neoadjuvant chemotherapy (NACT) is a central part of the comprehensive treatments for locally advanced malignant tumors and was first proposed by Frei in 1982 (1), referring to systemic chemotherapy applied before surgery or radiotherapy. NACT is superior in reducing the clinical tumor stage, increasing the surgical resection rate, reducing postoperative complications, preventing postoperative metastasis, and improving the postoperative survival rate of patients. Because of its sensitivity and efficiency, NACT is beneficial for establishing an effective chemotherapy regimen for postoperative adjuvant chemotherapy and for improving the long-term efficacy of treatment after surgery (2–9).

The tumor microenvironment (TME), which is closely associated with tumor progression and the response to treatments, includes blood vessels, immune cells, fibroblasts, and extracellular matrix (10–13). All immune components within the TME, including innate and adaptive immune cells, extracellular immune factors, and cell surface molecules, are defined as the tumor immune microenvironment (TIME), which is very relevant to the occurrence, development, recurrence, and metastasis of tumors (14–16). The immune structure, composed of the location, type, density, and functional status of immune cells in tumors, is different among patients and is crucial to the response rates and prognosis of patients (17–19).

Chemotherapy, a conventional treatment for most malignancies, can inhibit tumor cell mitosis and nucleic acid anabolism as well as directly interfere with tumor cell DNA replication (20). Although considered to weaken the immune system and induce various adverse effects in prior reports, recent studies have proven that classical cytotoxic drugs not only kill tumor cells but also induce immunogenicity. Meanwhile, chemotherapy activates the immune system by promoting lymphocyte activation and decreasing suppressive immune cells (21, 22).

NACT is a good clinical model to evaluate the effect of chemotherapy on the TIME because it is convenient to obtain paired tumor tissues before and after treatment. To provide strategies and theoretical bases for combining immunotherapy and enhancing the efficacy of NACT, this review summarizes the remodeling effect of NACT on the TIME in gastrointestinal tumors by outlining the changes in tumor-infiltrating immune cells before and after NACT, the efficacy prediction, and the associated prognostic value (Table 1).

**Table 1:** Summary of the remodeling effect of NACT on the TIME and the associated prognostic significance.

Cancer type	Markers	Changes with NACT	Efficacy prediction	Prognostic value	First author ^[Ref],^year
Gastric cancer	CD4 CD8 PD-1 PD-L1 TIM3	Increased	None	Improved OS with increased CD8, PD-1, and PD-L1	Yu Y (98), 2019
Gastric cancer	Foxp3	Unchanged	None	None	Yu Y (98), 2019
Gastric cancer	Foxp3	Decreased	None	None	Xing X (99), 2022
Gastric cancer	CD68	Decreased	None	None	Xing X (99), 2022
Gastric cancer	The diversity of TCR clonotypes	Increased	None	None	Xing X (99), 2022
Gastric cancer	CD68 CD163	Increased	None	Poor OS with increased CD163	Wei Q (100), 2021
Gastric cancer	DCs	Increased	None	Improved with increased DCs	Hu M (101), 2014
Gastric cancer	Foxp3	Decreased	None	Improved with decreased Tregs	Hu M (101), 2014
Gastric cancer	TANs	Decreased	Decreased TANs with tumor regression	None	Hoffmann A (102), 2021
Gastric cancer	B7-H4	Decreased	Decreased B7-H4 with tumor regression	Improved with decreased B7-H4	Maskey N (104), 2014
Gastric cancer	IL-17 CD8 Foxp3 Tbet CD20	Unchanged	None	None	Hennequin A (105), 2016
Gastric cancer	CD20	Decreased	None	None	Christina Svensson M (106), 2021
Esophagus cancer	CD4 CD8 HLA I	Increased	None	None	Tsuchikawa T (107), 2012
Esophagus cancer	PD-L1	Increased	None	None	Fukuoka E (108), 2019
Gastroesophageal junction adenocarcinoma	PD-L1	Increased	None	None	Jomrich G (109), 2022
Pancreatic cancer	HLA-I, HLA-II CD8	Increased	None	None	Michelakos T (111), 2021
Pancreatic cancer	Tregs M2	Decreased	None	Poor OS with increased M2	Michelakos T (111), 2021
Pancreatic cancer	Tregs MDSCs NK cells B cells	Decreased	None	Improved with decreased NK cells	Mota Reyes C (112), 2020
Pancreatic cancer	CD8 DCs	Increased	None	Improved with decreased DCs and CD4+ T cells	Mota Reyes C (112), 2020
Pancreatic cancer	PD-L1	Increased	None	None	Farren M R (114), 2020
Rectal cancer	CD8	Increased	Increased CD8^+^ T cells with tumor regression	None	Matsutani S (115), 2018
Rectal cancer	Ki67^high^ T cells	Increased	Increased Ki67^high^ T cells with tumor regression	Improved with increased Ki67^high^ T cells	Imaizumi K (116), 2020
Colorectal cancer	CD4^+^GzmB^+^ T cells	Increased	None	Improved OS and DFS with increased CD4^+^GzmB^+^ T cells	Qi J (117), 2021
Colorectal cancer	CD8 PD-L1	Increased	None	Improved DFS with increased T cells and PD-L1	Jary M (119), 2021
Liver metastases of colorectal cancer	CD8^+^ T cells	Increased	None	Poor prognosis with increased CD8^+^ T cells	Ledys F (123), 2018
Liver metastases of colorectal cancer	T cells	Increased within a short interval	None	None	Dagenborg V J (124), 2020
Liver metastases of colorectal cancer	MRC^+^CCL18^+^ M2	Decreased	Decreased MRC^+^CCL18^+^ M2 with tumor regression	None	Wu Y (122), 2022
Liver metastases of colorectal cancer	CTLs	Increased	Increased CTLs with tumor regression	None	Wu Y (122), 2022
Breast cancer	CD4 CD8 PD-1^+^CD8^+^ T cells CD73 PD-L1	Increased	None	None	Graeser M (128), 2021
Breast cancer	TILs	Increased after one cycle of NACT	Increased TILs and CD8^+^ T cells with tumor regression	None	Park Y H (129), 2020
Breast cancer	TILs	Decreased at the end of NACT	None	None	Park Y H (129), 2020
Breast cancer	M2	Increased at the end of NACT	None	None	Park Y H (129), 2020
Liver cancer	CD4^+^Foxp3^+^ T cells CD8^+^PD-1^+^ T cells	Decreased	None	Improved DFS with decreased CD4^+^Foxp3^+^ cells	Pinato D J (127), 2021
Oral squamous cell carcinoma	CD4 CD8 CD56	Increased	None	None	Takakura H (130), 2017
Oral squamous cell carcinoma	Tregs PD-1^+^ cells	Decreased	None	None	Takakura H (130), 2017
Non-small cell lung cancer	CTLs CD20^+^ B cells Tissue memory T cells	Increased	None	None	Gaudreau P O (131), 2021
Ovarian cancer	IFN-γ Th1 PD-L1	Increased	None	None	Böhm S (132), 2016
Ovarian cancer	Tregs	Decreased	None	None	Böhm S (132), 2016
Ovarian cancer	CD8^+^ Memory T cells	Unchanged	None	None	Böhm S (132), 2016
Ovarian cancer	TILs PD-L1	Increased	None	Improved PFS with increased TILs	Mesnage S J L (133), 2017
Ovarian cancer	CD8 CD3	Increased	None	Improved PFS with increased CD8^+^/Foxp3^+^, CD3^+^/Foxp3^+^, CD68^+^/CD163^+^ ratios	Leary A (134), 2021
Cervical cancer	CD200 CD4 CD8 CD20 CD56	Increased	None	None	Zhang Y (135), 2021
Osteosarcoma	CD3 CD8 PD-L1 Ki67^+^CD8^+^ T cells	Increased	None	None	Deng C (136), 2020
Osteosarcoma	MDSCs	Decreased	None	None	Deng C (136), 2020

### The composition of TIME with clinical significance

**Figure 1:**
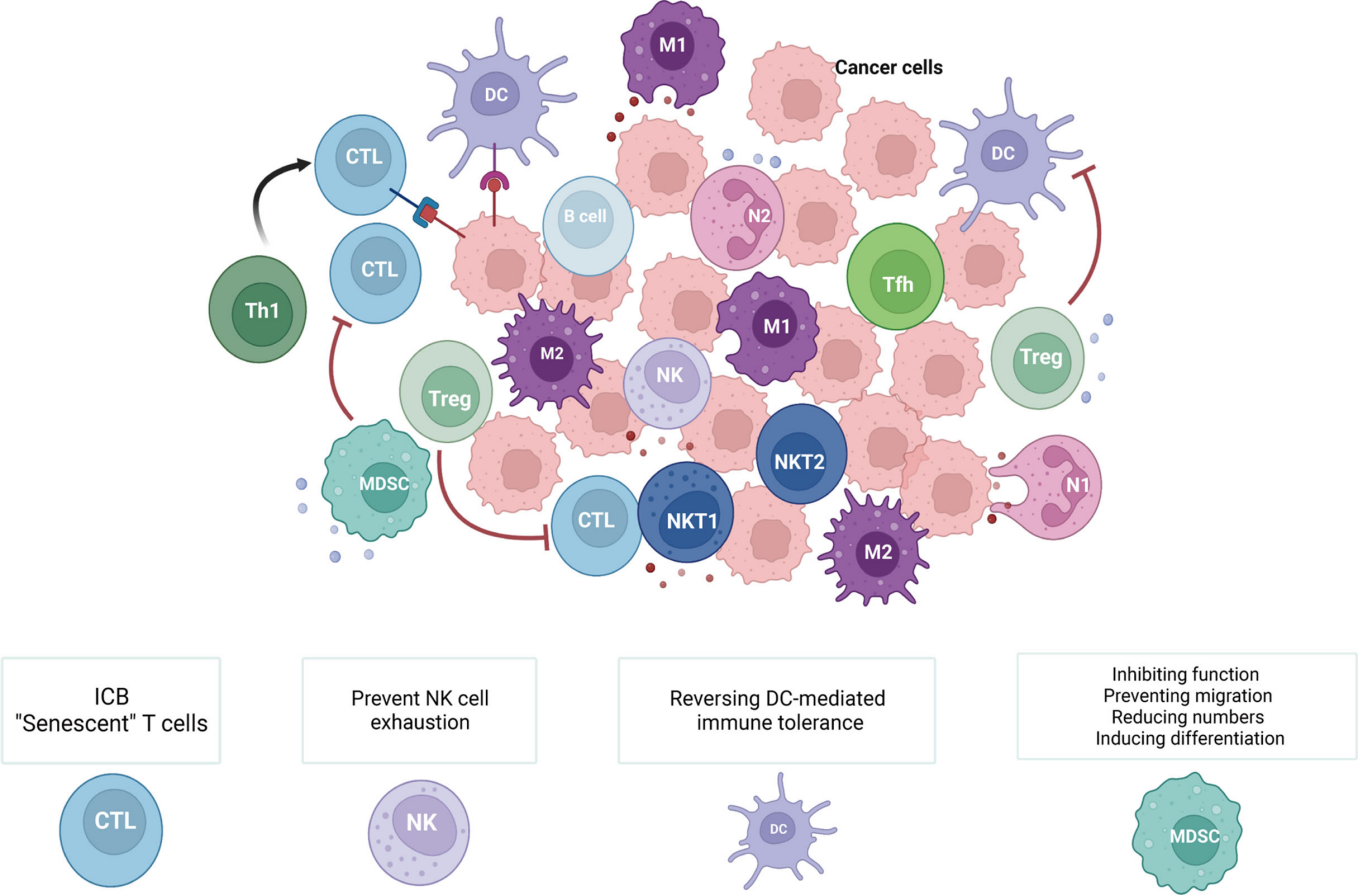
Immune cells within the TIME and related cancer immunotherapies. The TIME consists of various innate immune cells (macrophages, neutrophils, dendritic cells, myeloid-derived suppressor cells, and natural killer cells) and adaptive immune cells (T and B cells). During different stages of tumors and due to the diversity of the TIME, these immune cells have distinct roles in antitumor immunity. Therefore, methods for cancer immunotherapy that target tumor-suppressing immune cells include ‘exhausted’ T cells (ICB), ‘senescent’ T cells, exhausted NK cells, DC-mediated immunological tolerance, and MDSCs (inhibiting the function of MDSCs, preventing the migration of MDSCs, reducing the number of MDSCs, and inducing the differentiation of MDSCs). (Created with Biorender.com).

The TIME, where tumor cells can effectively modify their surroundings by secreting a variety of cytokines and chemokines, is an integral and indispensable part of tumor tissues (**Figure 1**). Moreover, immune cells in the TIME have been proven to be related to tumor development, metastasis, and recurrence. Innate immune cells (macrophages, neutrophils, dendritic cells, myeloid-derived suppressor cells, and natural killer cells) and adaptive immune cells (T and B cells) in the TIME can act as tumor antagonists or tumor agonists. Although these immune cells tend to kill tumor cells in the early stage, tumor cells are still able to evade immune surveillance through a variety of mechanisms, even creating a variety of ways to inhibit the function of antitumor immune cells and reduce the clinical effectiveness of antitumor therapies.

#### CD8^+^ T cells

CD8^+^ T cells, a key component of the adaptive immune system (23), play an important role in antitumor immune responses by recognizing and eliminating tumor cells. It has been reported that the density of CD8^+^ T cells is closely related to the clinical prognosis of patients (24, 25). To elicit efficient anticancer immune responses, CD8^+^ T cells need to go through a series of events in the ‘cancer immune cycle’, including neoantigen production by tumor cells, antigen recognition and antigen presentation by dendritic cells (DCs), and activation of cytotoxic lymphocytes (cytotoxic T lymphocytes, CTLs). Finally, activated CTLs infiltrate into tumor tissues and destroy tumor cells (26–28).

However, the immunosuppressive network in the TME can remodel CD8^+^ T cells, leading to CD8^+^ T-cell dysfunction, failure to remove tumor cells, and weakened antitumor immunity (26, 29). Two of the main dysfunctional states of T cells in the TME are ‘exhaustion’ and ‘senescence’ (29, 30). ‘Exhausted’ T cells are characterized by upregulated expression of inhibitory receptors, such as programmed cell death protein 1 (PD-1), cytotoxic T-lymphocyte antigen-4 (CTLA-4), T-cell immunoglobulin and mucin domain containing-3 (Tim-3), lymphocyte activation gene 3 (LAG-3), T-cell immunoreceptor with Ig and ITIM domains (TIGIT), and so on (29, 31). Elevated levels of ‘exhausted’ T cells in tumors are associated with a poor prognosis, and targeting inhibitory receptors to restore CD8^+^ T-cell function has important implications in clinical treatments (29, 32). Moreover, the efficacy of immune checkpoint blockade (ICB) therapy has been demonstrated in various antitumor immunotherapies (29, 33). Unlike ‘exhausted’ T cells, ‘senescent’ T cells exhibit a senescence-associated secretory phenotype (SASP), producing a mass of inflammatory cytokines (IL-2, TNF-α, IFN-γ) and suppressive cytokines (IL-10 and TGF-β). Due to their vital roles in immunosuppression and tumor progression, senescent T cells are new targets for antitumor immunotherapy (29, 34).

#### CD4^+^ T cells

CD4^+^ T cells, helper T lymphocytes (Th), are involved in adaptive immune responses (35) and can regulate the state and function of other immune cells, playing a significant role in autoimmunity, allergic reactions, and antitumor immune responses (36). CD4^+^ Th cells are divided into different subpopulations, including Th1, Th2, Th17, Tfh, and Tregs, and each subpopulation displays a specific role in tumor immune responses, inhibiting or promoting tumor cell growth (24). After antigen-induced activation, Th1 cells generate inflammation by producing inflammatory factors such as TNF-α and IFN-γ, as well as promoting DC maturation and improving CTL function (37). Th1 cells are frequently associated with favorable clinical outcomes in patients (38). Studies have shown that cytokines such as IFN-γ secreted by Th1 cells not only enhance CD8^+^ T-cell differentiation but also directly inhibit tumor cell growth by causing their senescence (39, 40). Tfh (follicular helper T cells, Tfh) cells exert antitumor effects in tumors by mediating B-cell differentiation into plasma cells, antibody class switching, and the production of antibodies. Although the presence of Tfh cells predicts a favorable outcome, their antitumor functions may be blocked by the PD-L1/PD-1 signaling pathway in the TME (41).

Regulatory T cells (Tregs), expressing forkhead protein P3 (Foxp3), are pivotal in maintaining immune homeostasis and peripheral tolerance (42), as well as suppressing overactive immune responses such as autoimmune diseases (43). Clonal expansions of Tregs are highly heterogeneous, observed in many types of tumors, and are closely associated with a poor prognosis and reduced survival rates (44, 45). In the TIME, Tregs mainly inhibit antitumor immune responses through two mechanisms and participate in the process of immune escape (43, 46). Tregs release inhibitory cytokines (such as IL-10 and TGF-β) to prevent the infiltration and activity of tumor-specific T cells (37). However, Tregs also impede the development and maturation of DCs (47). Tumor-infiltrating Tregs express numerous negative costimulatory molecules (such as PD-L1 and PD-L2) that inhibit CD8^+^ T-cell activation and interact with the receptor PD-1 on CD8^+^ T cells to block TCR signaling, thereby repressing the activity of CD8^+^ T cells (48).

#### NK cells and NKT cells

NK (natural killer, NK) cells are innate lymphocytes that kill tumor cells nonspecifically in the early stages, performing antitumor immune surveillance (49). According to their expression of CD16 and CD56, NK cells are divided into two subsets: CD56^hi^CD16^±^ and CD56^lo^CD16^hi^ (50). The CD56^hi^CD16^±^ subset secretes inflammatory cytokines, whereas CD56^lo^CD16^hi^ mainly exerts cytotoxic and killing functions (50). To promote antitumor immune responses, NK cells recruit DCs to tumor sites and secrete cytokines, promoting DC maturation (51). In addition, the quantity of infiltrating NK cells is remarkably linked to improved patient outcomes (52, 53). Tumor cells and other cells in the TME may prevent NK-cell activation and inhibit their function by secreting a variety of cytokines (including IL-6, IL-10, TGF-β, PGE2, and IDO) (49, 54). Moreover, the activation of inhibitory immune checkpoints (such as CTLA-4, PD-1, and Tim-3) hampers NK-cell function (49). Blocking the checkpoint receptor TIGIT can prevent NK-cell exhaustion and restore NK-cell activity (55).

NKT (natural killer T, NKT) cells, a subset of natural lymphocytes, simultaneously express certain surface markers of T cells and NK cells (56) and play a critical role in innate and adaptive immunity. In light of the various types of TCRs, NKT cells exhibit two main subtypes: type I NKT and type II NKT cells. Type I NKT cells secrete Th1-type cytokines (IFN-γ, IL-12) to boost antitumor immune responses (57), while type II NKT cells suppress antitumor immunity by producing IL-13 to restrain the function of CD8^+^ T cells (58). In addition, NKT cells transform into their various subpopulations and exhibit different functions in different environments (59, 60).

#### Dendritic cells

DCs are recognized as the most powerful and specialized antigen-presenting cells known so far (61). DCs recognize and present antigens to CTLs and simultaneously provide costimulatory signals and cytokines to T cells for further activation (49, 62). As reported, infiltrating DCs in the TME are positively associated with the prognosis of patients (63, 64). DCs are functionally differentiated into distinct subpopulations: classical DCs (cDCs), plasmacytoid DCs (pDCs), and monocyte-derived inflammatory DCs (moDCs) (59). cDCs and pDCs are present and active under steady-state conditions, whereas moDCs appear only during inflammation (59). In the TME, multiple immunosuppressive factors, such as VEGF and IL-10, hinder DC maturation and impair their capability for antigen presentation and T-cell activation (65, 66). Tumor cells can also polarize DCs into tolerogenic DCs (67), and reversing DC-mediated immune tolerance is the main therapeutic strategy against DCs in the TME.

#### Macrophages

Macrophages derived from circulating monocytes are significant immune cells in the TME, accounting for approximately 50% of tumor tissues (68–70). Macrophages play a principal role in immune defense, immune homeostasis, immune surveillance, antigen presentation, and immune regulation (71). Macrophages exhibit two distinguished types, inflammatory M1 (classically activated) and immunosuppressive M2 (alternately activated) (72). M1 macrophages secrete inflammatory cytokines and reactive oxygen/nitrogen that are essential for host defense and tumor killing (73, 74). Monocytes in the bone marrow are recruited into the TME under the action of various chemokines and further differentiate and develop into mature macrophages (75–77), tumor-associated macrophages (TAMs). TAMs are phenotypically and functionally similar to M2 macrophages, which express low levels of MHC class II molecules, manifest decreased antigen presentation activity, and secrete high levels of immunosuppressive cytokines. During the course of tumor evolution, TAMs accelerate tumorigenesis, development, invasion, and metastasis, especially in angiogenesis and lymphangiogenesis (72, 78).

#### Neutrophils

Neutrophils represent the front line of the body’s defense system, accounting for 55% to 70% of circulating leukocytes (79). By creating neutrophil extracellular traps and secreting different cytokines and chemokines, neutrophils not only fight infection by phagocytosis but also trigger inflammatory responses (80, 81). Tumor-associated neutrophils (TANs) manifest N1 (tumor-suppressing) and N2 (tumor-promoting) phenotypes, and the phenotype of TANs depends on the type and stage of tumors (59). In the early stage of tumorigenesis, neutrophils secrete cytokines (such as IFN-γ) to recruit and stimulate immune cells to restrain tumor growth. Neutrophils isolated from early-stage lung cancer prompted CD4^+^ T cells to release IFN-γ, which in turn enhanced the differentiation of CD8^+^ T cells (82) and supported antitumor immunity. However, with the development of tumors, neutrophils gradually shift into an immunosuppressive phenotype (59, 83) and promote tumor progression (82). TANs have been reported to be associated with a poor prognosis in gastric cancer by enhancing the migration, invasion, and epithelial-mesenchymal transition of gastric cancer cells (84). Additionally, TANs recruit macrophages and Tregs to the TME, resulting in liver cancer progression and resistance to sorafenib (14).

#### Myeloid-derived suppressor cells

MDSCs, tumor-promoting cells in the TME, are bone marrow-derived immunosuppressive heterogeneous cell populations consisting of myeloid progenitor cells, immature macrophages, immature granulocytes, and immature dendritic cells (85). Granulocytic or polymorphonuclear MDSCs (PMN-MDSCs) and mononuclear MDSCs (monocytic MDSCs, M-MDSCs) are the two main subsets of MDSCs (86, 87). In addition to promoting tumor angiogenesis, stemness, and metastasis, activated MDSCs suppress antitumor immunity mediated by T cells, NK cells, and macrophages (88, 89). In addition, MDSCs are linked to a shorter overall survival (50) and they are vital therapeutic targets due to their important role in the establishment of a premetastatic niche (59). Currently, therapies against MDSCs basically focus on inhibiting their immunosuppressive function, preventing their migration to the TME, reducing their numbers, and driving their differentiation into an inflammatory phenotype (90).

#### B cells

Although the research to date primarily concentrates on T cells, increasing evidence suggests that tumor-infiltrating B lymphocytes (TIL-Bs), comprising tumor-infiltrating B cells and plasma cells, have an indispensable synergistic role in tumor control (91). The most prominent phenotypes of TIL-Bs are the effector and regulatory B-cell (Breg) subsets (91). Depending on the composition of the TME and the phenotype and antibodies produced by B cells, TIL-Bs exert antitumor or protumor effects (92). By presenting antigens to CD4^+^ and CD8^+^ T cells, B cells trigger antigen-specific immune responses in the TME (92, 93). In addition, B cells promote tumor-specific B-cell maturation and isotype switching and tumor-specific T-cell effects by promoting the formation of tumor-associated tertiary lymphoid structures (TLSs) (92, 94). *In vitro*, B cells isolated from melanoma patients triggered antibody-dependent cell cytotoxicity (ADCC) after producing IgG (immune globulin G, IgG), thus eliminating tumor cells (92). Studies have shown that the presence of TIL-Bs is associated with a favorable prognosis in patients (91, 95). However, Bregs can suppress antitumor immunity by releasing inflammatory mediators (such as IL-10) and expressing inhibitory costimulatory molecules (such as PD-L1), and the infiltration of Bregs is positively correlated with Tregs (96, 97).

### The remodeling effect of NACT on the TIME and its prognostic value in gastrointestinal tumors

#### Gastric cancer

An analysis of paired pre- and post-NACT tumor samples from 60 patients with gastric cancer revealed that, whereas the expression of Foxp3 remained unchanged, the expression of CD4, CD8, PD-1, PD-L1, and TIM3 increased dramatically after NACT. These increased CD4^+^ T cells and CD8^+^ T cells following NACT demonstrated that chemotherapy sparked hosts’ antitumor immune responses. However, following initial immune stimulation, IFN-γ produced by activated T cells generated negative feedback to induce the expression of PD-L1, which then reprogrammed the TIME from an active state to a generally balanced environment. This indicated the dynamic bidirectional transfer of the gastric cancer TIME during chemotherapy (98). Additionally, variations in TIM3, PD-1, and PD-L1 between baseline and post-NACT demonstrated a substantially positive relationship with one another, suggesting that dual targeting against PD-L1 and TIM3 may be a potentially beneficial choice for gastric cancer patients (98). In multivariate analysis, upregulated expression of CD8^+^ T cells, PD-1, and PD-L1 post-NACT were favorable prognostic factors of overall survival (OS) (98), possibly because they reflected powerful immunological flexibility.

However, another study uncovered slightly dissimilar outcomes. Foxp3^+^ Tregs decreased significantly in a cohort of 30 matched patients (before and after NACT), according to research by Xing X et al. Furthermore, patients who responded to chemotherapy had higher levels of Tregs prior to NACT, and following NACT, these patients had lower Treg and higher CTL levels (99). In addition, the TCR numbers decreased significantly, while the number of specific TCR clones increased, indicating an increase in T-cell diversity following NACT (99). This suggests that chemotherapy can boost antitumor immunity and alleviate immunosuppression in gastric cancer patients by increasing the expansion of T cells. Moreover, Xing X et al. also analyzed another 1416 patients (341 of whom underwent NACT) and discovered that the recruitment of CD68^+^ macrophages was reduced in the NACT group and that CD8^+^ T cells post NACT were an independent predictor of a better outcome (99).

In contrast, the study by Wei Q et al. showed that in 50 paired patients, the expression of CD68 and CD163 increased significantly after NACT, implying that NACT promoted the recruitment of CD68^+^ macrophages and CD163^+^ macrophages (100). Higher CD163 expression after NACT was found to be an independent predictor of a poor OS in multivariate analysis. Moreover, the CD163/CD68 ratio and the CD8/CD3 ratio after chemotherapy were correlated, and a higher CD8/CD3 ratio neutralized the immunosuppressive effect of M2 macrophages (100).

Another examination of postoperative samples from 102 patients (56 patients receiving NACT: NACT group) revealed that DCs increased considerably in the NACT group and were significantly associated with histopathological type, depth of invasion, TNM stage, and lymph node metastasis. Consistent with the above study (99), this study also noted a reduced number of Tregs following NACT. Increased DCs and decreased Tregs post-NACT served as biomarkers for a favorable prognosis in gastric cancer (101).To explore the alterations of TANs in the TME, Hoffmann A et al. analyzed postoperative gastric cancer tissues from 622 patients (173 patients receiving NACT) and assessed TANs in the mucosa, tumor surface, tumor center, invasion front, and tumor scar. According to their findings, TANs at the invasion front decreased remarkably in the NACT group, and the proportions of TANs at the tumor center and invasive front were correlated with tumor regression. TANs were also linked to the ratio of CD8^+^ T cells in the tumor center and invasive front, and a higher density of CD8^+^ T cells in the tumor center was associated with an improved OS (102). The decreased proportion of TANs post-NACT was further confirmed by transcriptome sequencing data from another study involving 35 paired patients (103). These studies demonstrate that the systemic influences of NCAT (leukopenia and neutropenia) also lead to local effects of markedly reduced TANs in the TME (102).

In addition to concentrating on tumor-infiltrating immune cells, studies have also investigated the expression of costimulatory molecules after NACT. The expression of the negative costimulatory molecule B7-H4 decreased after NACT, and its lower expression was a biomarker for treatment effectiveness and a good prognosis. Thus, NACT enhanced antitumor immunity by downregulating the expression of the negative costimulatory molecule B7-H4, leading to reduced survival rates in gastric cancer patients (104).

Despite the fact that the bulk of research has shown variations in the TIME, Hennequin A et al. found no significant variances. In their research, the proportions of IL-17^+^ T cells, CD8^+^ T cells, Foxp3^+^ Tregs, Tbet^+^ T cells, and CD20^+^ B cells in the central and invasive margins of postoperative tumor tissue from 82 patients (42 of whom received NACT) were analyzed. There was no discernible impact of NACT on immune cell density, kind, or prognostic value (105). In contrast, another study found a reduction in the fraction of CD20^+^ B cells after NACT in 39 esophageal and 109 gastric cancer paired patients (pre-NACT and post-NACT) (106).

In view of the differences in the antibodies used, the number of samples, and the heterogeneity between patients and within the tumor, there are some discrepancies among the aforementioned studies. In summary, NACT modulates the tumor immunity of gastric cancer in a bidirectional manner, not only stimulating antitumor immune responses but also inducing tumor immunosuppression. The effect of chemotherapy on the TIME should be taken into account when devising combined regimens.

#### Esophageal cancer

In an analysis of 18 patients with esophageal squamous cell carcinoma (8 patients receiving NACT: NACT group), Tsuchikawa T et al. illustrated that CD4^+^ T cells, CD8^+^ T cells, and the expression of HLA I increased in the NACT group and that in esophageal squamous cell carcinoma, NACT improved patients’ survival by inducing T lymphocyte infiltration and upregulating HLA class I expression (107).

Similarly, another study showed that in 69 paired patients (pre- and post-NACT), the infiltration of CD8^+^ T cells and the expression of PD-L1 on immune cells increased following NACT, which suggested that PD-1/PD-L1 blockade synergizes with NACT in the treatment of patients with esophageal squamous cell carcinoma (108). Increased levels of PD-1 were also observed in 40 patients with gastroesophageal junction adenocarcinoma after NACT (109).

Overall, NACT influences the TIME of esophageal cancer and gastroesophageal junction tumors, and combined therapy targeting the PD-1/PD-L1 pathway may improve the prognosis of these patients.

#### Pancreatic cancer

An investigation of the gene expression of pancreatic ductal adenocarcinoma (PDAC) in the GEO database (gene chip: GSE129492) made it obvious that 83 genes were differentially expressed between the NACT and non-NACT groups. Furthermore, these genes were mainly engaged in the following biological pathways: immune system, cytokine signal transduction in the immune system, innate immune system, TCR signaling in CD8^+^ T cells, CD40/CD40 L signaling, and TCR signaling in CD4^+^ T cells. These findings suggest that NACT affects the TIME of PDAC (110).

Michelakos T et al. analyzed the expression of HLA-I, HLA-II, and immune cells from 248 patients with PDAC. They found that after NACT, HLA-A deficiency was reduced, immune escape of tumor cells was weakened, the density of CD8^+^ T cells increased, and Tregs and M2 macrophages decreased. Additionally, abundant infiltration of M2 macrophages was an independent predictor of a poor OS. Therefore, NACT ameliorated the immunosuppressive TIME in PDAC (111).

Similar to the study mentioned above (111), another examination involving 27 pancreatic cancer patients treated with NACT substantiated that NACT reactivated a potent and prolonged local immune response in PDAC. After NACT, the infiltration of Tregs, MDSCs, NK cells, and B cells was reduced significantly, while the proportion of CD8^+^ T cells to all leukocytes and the percentage of DCs increased dramatically. Furthermore, CD4^+^ T cells and NK cells were independent prognostic factors (112). Identical outcomes were noticed in another investigation where the function of Tregs and B cells was suppressed following NACT in PDAC (113).

Research has also been performed on the changes in Tfh cells in PDAC following NACT. As previously reported, the PD-L1/PD-1 signaling pathway may suppress the antitumor activity of Tfh cells (41). In patients who received NACT, the function of Tfh cells was reversed, and their capacity to express CXCL13 and IL-21 was considerably enhanced. As a result, Tfh cells shaped an immune-active TIME by recruiting CD8^+^ T cells and promoting the maturation of B cells into antibody-producing plasma cells. Meanwhile, a higher Tfh cell density was associated with a better patient prognosis. These results suggested that NACT unleashed antitumor immunity locally in PDAC by reversing Tfh cell function (41).

In addition, NACT increased the expression of the costimulatory molecule PD-L1 in 24 PDAC patients (6 of whom received NACT) (114), suggesting that NACT patients will benefit from PD-1/PD-L1 targeted therapy.

On the whole, NACT can reverse the suppressive microenvironment of pancreatic cancer and reinforce patients’ antitumor immune responses. Incorporating immunotherapy (such as PD-1/PD-L1 targeted therapy) may be beneficial for patients with pancreatic cancer.

#### Colorectal cancer

According to an analysis by Matsutani S et al. of tumors from 64 rectal cancer patients (33 of whom received NACT), CD8^+^ T cells increased following NACT, and a higher density of CD8^+^ T cells was associated with better clinicopathological responses, which demonstrated that T-cell-mediated immune responses play an essential role in the clinicopathological reaction to NACT (115). Additionally, detection of T-cell activation status revealed increased T-cell activation after NACT. T-cell subset characterization of 188 rectal cancer patients (46 patients receiving NACT) showed that the density of total and activated T cells (Ki67^high^) increased remarkably after NACT, and the infiltration of T cells was much greater in patients with a better therapeutic outcome. In the multivariate analysis, the number of stromal Ki67^high^CD8^+^ T cells following NACT was a better prognostic factor (116).

NACT can also activate local immune responses in colorectal cancer. After analyzing immune cells in 77 patients with stage II/III colorectal cancer (38 patients receiving NACT), researchers discovered that in the NACT group, the infiltration of CD4^+^GzmB^+^ T cells in the central region of the tumors increased, and CD4^+^GzmB^+^ T cells were a better prognostic factor for OS and disease-free survival (DFS) (117). Furthermore, several studies noted increased expression of CD8^+^ T cells and PD-L1 in tumors (118) and metastatic sites (119, 120) of colorectal cancer after NACT, with upregulated T cells and PD-L1 on immune cells predicting a better DFS (119). Therefore, NACT enhanced the antitumor immune response by promoting the recruitment of CD4^+^GzmB^+^ T cells and CD8^+^ T cells in colorectal cancer. Recruited CD8^+^ T cells then released IFN-γ, which in turn led to upregulated PD-L1 levels.

Liver metastases, the leading cause of colorectal cancer death, occur in 50% of patients and manifest a highly heterogeneous microenvironment (121, 122). Understanding how NACT affects the microenvironment of liver metastasis is essential for developing therapeutic approaches as well as for determining the key mechanisms of NACT. One study, comprising 114 patients with colorectal cancer liver metastasis (47 patients receiving NACT), highlighted that T-cell infiltration and PD-L1 expression increased significantly in patients who accepted NACT (123). These results suggested that NACT recruited immune cells in patients with colorectal cancer liver metastases, but the recruited CD8^+^ T cells might induce immune tolerance, which is responsible for the poor outcome of certain patients. Another study found time-dependent alternations in T cells, with T cells increasing in the group that underwent surgical resection only within a short interval (<9.5 weeks) after the completion of NACT (124). Moreover, the Treg/CTL ratio was lower in the short-interval group (124). This study illustrated that the immunosuppressive milieu gradually becomes prominent after NACT, while the time-dependent changes in immune cells indicated that there might be a time window of opportunity for application of immunotherapy.

Wu Y et al. depicted the spatiotemporal immune landscape of colorectal cancer liver metastases at the single-cell level (a total of 20 patients, 11 of whom received NACT) and learned that there were highly metabolically activated MRC^+^CCL18^+^ M2 macrophages in the metastatic milieu. In patients with partial remission, MRC^+^CCL18^+^ M2 macrophages decreased and CTLs increased in the TIME of liver metastasis, indicating that antitumor immunity had recovered, whereas the inhibitory TIME was more obvious in nonresponding patients (122). This study revealed that NACT potently restored the tumor immunological homeostasis in patients who responded to chemotherapy, and targeting metabolic pathways can be employed as a combined therapy option.

To conclude, in patients with rectal cancer and colorectal cancer, NACT stimulates antitumor immune responses, and combining immunotherapy may be an effective choice. Meanwhile, NACT reshapes the TIME of colorectal cancer liver metastases in a time-dependent manner, and it is of benefit for these patients to combine immunotherapy or metabolic therapy at an appropriate time point.

#### Liver cancer

Transarterial chemoembolization (TACE) is a preferred local therapy for patients with Barcelona stage B liver cancer (125) and is able to hinder tumor progression. As has been reported, the efficacy of TACE is correlated with treatment-induced immune modulation (126). After analyzing 119 patients with liver cancer (58 of whom received TACE before surgery), Pinato D J et al. observed that CD4^+^Foxp3^+^ cells and CD8^+^PD-1^+^ cells were lower in the TACE group, and lower CD4^+^Foxp3^+^ cells were associated with a better DFS. In addition, signaling pathways associated with chemokine secretion, regulation of immune cell function, complement cascade activation, and production of interleukins and cytokines were upregulated. This study found that TACE has pleiotropic effects in regulating the TIME, reducing the proportions of Tregs and exhausted T cells, and upregulating proinflammatory signaling pathways. Adding immunotherapies such as depleting Tregs and inhibiting Treg function to enhance the antitumor effect of TACE can be viable therapeutic strategies (127).

#### Other types of tumors

There are studies regarding early changes in immune infiltration post NACT in breast cancer. A study involving 66 paired triple-negative breast cancer patients showed that after one cycle of NACT, the infiltration of total T cells, CD4^+^ T cells, CD8^+^ T cells, and PD-1^+^CD8^+^ T cells increased, and the expression of CD73 and PD-L1 also increased significantly (128). Another study also displayed dynamic changes in the TME following NACT. According to the study by Park YH et al., NACT induced dynamic changes in the TME, and the role varied with breast cancer subtypes and pathological remission. Only one cycle of NACT increased the infiltration of TILs and induced an activated immune microenvironment. Compared with the baseline, the residual tumors exhibited an immunosuppressive state at the end of treatments, in which the abundance of TILs and immune-stimulated cell types was reduced, and immunosuppressive M2 macrophages were increased. Higher levels of post-NACT TILs and CD8^+^ T cells were associated with complete pathological remission. Additionally, the on-treatment immune response was more predictive of treatment outcome than immune signatures in paired baseline samples, although these were strongly correlated (129). This study showed that NACT initially induced antitumor immunity, but eventually, it became immunosuppressive, and the antitumor efficacy was impaired. Including immunomodulatory therapy might be more beneficial early on rather than later (129).

An analysis of 18 patients with oral squamous cell carcinoma (8 of whom received NACT) showed that the infiltration of CD4^+^ T cells, CD8^+^ T cells, and CD56^+^ NK cells into tumors were much greater, whereas the proportions of Tregs and PD-1^+^ cells decreased. These results support the immunomodulatory role of NACT in oral squamous cell carcinoma (130).

Gaudreau P O et al. studied the TIME of 511 patients with non-small cell lung cancer (146 of whom had NACT) and discovered that following NACT, the infiltration of CTLs, CD20^+^ B cells, CD8^+^CD103^+^ and CD4^+^CD103^+^PD-1^+^TIM3^-^ tissue memory T cells increased noticeably. NACT, however, had no impact on the clonality and abundance of TCRs or tumor mutational load. This study showed that NACT promoted antitumor immunity in non-small cell lung cancer by recruiting T and B cells and by phenotypic polarization toward cytotoxic and memory CD8^+^ T cells or CD4^+^ memory T cells, suggesting that combining T-cell agonists (such as TLR9, STING, and IL-10 agonists) can be a promising treatment for non-small cell lung cancer (131).

NACT reshaped the TIME of ovarian cancer as well. An analysis of 54 patients with high-grade serous ovarian cancer who received NACT showed that IFN-γ produced by CD4^+^ T cells and antitumor Th1-related genes increased after NACT, whereas CD8^+^ T cells and CD45RO^+^ memory cells remained unaltered. Moreover, the expression of PD-L1 was significantly elevated, and in patients responding well to chemotherapy, the proportion of Tregs was reduced (132). Similarly, Mesnage S J L et al. also observed a significant increase in TILs and PD-L1 following NACT, and multivariate analysis showed that a higher TIL level post NACT was an independent predictor of a better PFS (133). More recently, the study by Leary A et al. focused on the impact of NACT on the balance between immune-active and immune-tolerant subpopulations (134). Their results indicated that NACT significantly increased CD3^+^ T cells and CD8^+^ T cells, whereas higher CD8^+^/Foxp3^+^, CD3^+^/Foxp3^+^, and CD68^+^/CD163^+^ ratios after NACT were associated with a better PFS (134). Altogether, NACT can enhance the immune response by regulating the balance between immune-active and immune-tolerant subsets, but this effect is attenuated by elevated PD-L1. Chemotherapy combined with immunotherapy can help to improve disease control in advanced high-grade serous ovarian cancer (132–134).

In a retrospective analysis of 109 patients with cervical cancer, increased signaling in CD4^+^ T cells, CD8^+^ T cells, CD20^+^ B cells, and CD56^+^ NK cells was noticed after NACT, particularly in those who had a good response. By RNA sequencing, upregulation of the immunosuppressive molecule CD200 was also detected. This study suggested that NACT improved local antitumor immunity in cervical cancer, and immune checkpoint inhibitors are worthy of further exploration as an addition to NACT therapy (135).

Deng C et al. showed that after NACT, CD3^+^ T cells, CD8^+^ T cells, Ki67^+^CD8^+^ T cells, and PD-L1^+^ immune cells increased, while HLA-DR-CD33^+^ MDSCs decreased. The conclusion was drawn that NACT activated the local immune state of osteosarcoma and relieved the immunosuppressive state, and research into the immunomodulatory effect of NACT on patients with osteosarcoma may offer a stronger theoretical foundation for selecting immunotherapies to combine with NACT (136).

## Conclusion and perspectives

The TIME plays a significant role in the survival and prognosis of patients. Researchers have demonstrated that chemotherapy can alter the TIME by regulating the amount and activity of various immune cells. In light of its convenience in obtaining clinical samples before and after chemotherapy, extensive research has focused on the effect of NACT on the TIME in gastrointestinal cancer, which assists in offering a reference for formulating more effective combined therapeutic methods. In this review, we recapitulated the remodeling effect of NACT on the TIME, the efficacy prediction role of infiltrating immune cells, and their associated prognostic value in multiple tumors (**Table 1**). In general, NACT can impact the TIME and improve antitumor immunity by changing the number and function of infiltrating immune cells. After NACT, antitumor immune cells, such as CD4^+^ T cells, CD8^+^ T cells, M1 macrophages, and DCs, increase, while immunosuppressive cells tend to decrease. Additionally, NACT can alter the expression of inhibitory receptors such as PD-1 and CTLA-4, and therapies combining ICB with NACT may be a promising approach to improving the treatment response and survival of patients.

There are, however, some defects in the current research that need to be resolved immediately. First, the fundamental status of the included populations, the severity of their disease, and the number of cycles of chemotherapy, are quite different across studies. In addition, standards and norms for the score of tumor-infiltrating immune cells vary from research to research. Finally, inadequate preoperative biopsy representativeness and inconsistent evaluation time points also lead to discrepancies. To minimize these biases and better elucidate the immune alterations, we need to broaden the included cohorts and set precise criteria for assessing the infiltrating immune cells. Nevertheless, the effort is worth it due to the expectation that appropriate individualized immunotherapies will be administered based on the post-NACT tumor-infiltrating immune cell profile, which is expected to become a new approach to antitumor immunochemotherapy in the future.

## Author contributions

YW, PG, ZH, and LC conceived the present study. XL, YJ, JYL, JL and YZ conducted the literature search. The first draft of the manuscript was written by YW and PG. XP, BN, and XZ critically revised the work. All authors contributed to the article and approved the submitted version.

## Funding

This study was supported by grants from the National Natural Science Foundation of China (82072707), the Scientific Research Program of the Shanghai Municipal Commission of Science and Technology (19411970700 and 20Y11909400) and the Changhai Hospital 234 Project (2019YXK019 and 2020YXK029).

## Conflict of interest

The authors declare that the research was conducted in the absence of any commercial or financial relationships that could be construed as a potential conflict of interest.

## Publisher’s note

All claims expressed in this article are solely those of the authors and do not necessarily represent those of their affiliated organizations, or those of the publisher, the editors and the reviewers. Any product that may be evaluated in this article, or claim that may be made by its manufacturer, is not guaranteed or endorsed by the publisher.
